# Case report: Interleukin-17 targeted biological therapy in netherton syndrome

**DOI:** 10.3389/fped.2023.1297658

**Published:** 2023-11-29

**Authors:** Rahul Mahajan, Shirin Bakshi, Anoop Kumar, Dipankar De, Sanjeev Handa

**Affiliations:** Department of Dermatology, Venereology & Leprology, Postgraduate Institute of Medical Education and Research, Chandigarh, India

**Keywords:** netherton syndrome, secukinumab, IL-17, ichthyosis, genodermatology

## Abstract

Netherton syndrome (NS) is rare and multisystemic congenital skin disorder classically distinguied as a triad of congenital ichthyosiform erythroderma, trichorrhexis invaginata (TI), and an atopic diathesis. Recent advances in pathogenesis have explored the role of IL-23/Th17 pathway in NS. Herein, we present a 17 years old girl harbouring homozygous four base pair deletion in exon 26 of the *SPINK5* gene, presented with pruritus, scaling, dry skin and generalized eczematous lesions. She was administered anti IL17A (subcutaneous secukinumab) therapy. The treatment was well tolerated and resulted in a favourable clinical response, reduction of the *IL17A* gene expression and CD4 + Th17 cell population after 6 months which revealed an abrogation of Th17-skewing during therapy.

## Introduction

Netherton syndrome (NS) is a difficult to treat autosomal recessive disorder that is associated with significant morbidity. It is characterised by the triad of congenital ichthyosiform erythroderma or ichthyosis linearis circumflexa, hair shaft abnormalities, and atopic diathesis. There is a paucity of therapeutic options that can lead to a satisfactory response in patients with NS. Recent studies have highlighted the possibility of targeting the interleukin-17 driven inflammatory pathway as an effective treatment strategy for the treatment of NS. Here, we present a case of NS who was successfully treated with secukinumab.

## Report of a case

**Figure 1:**
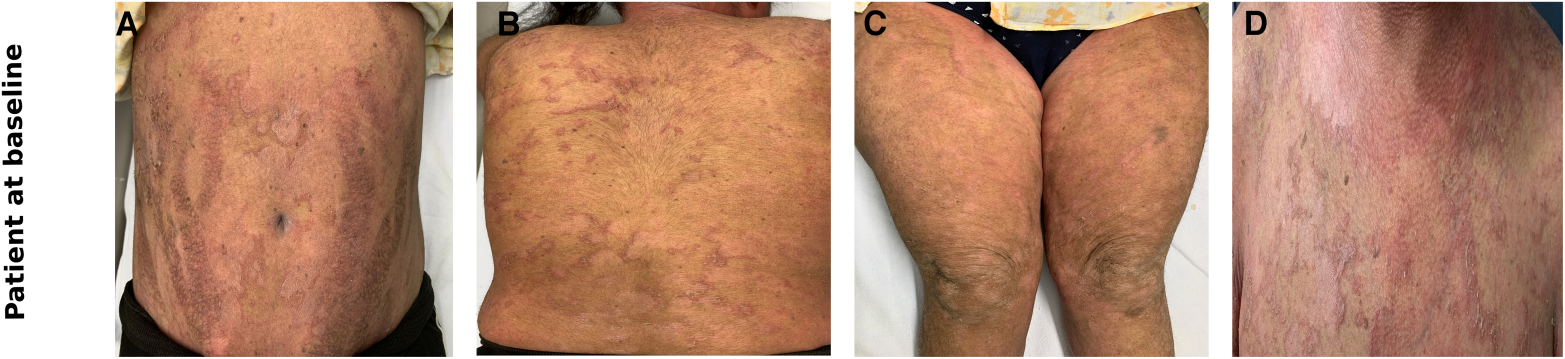
Clinical photographs of the patient at baseline showing erythema and ichthyosis linearis circumflexa like scales over the abdomen (**A**), back (**B**), thighs (**C**), chest (**D**).

A 17-year-old girl, known case of NS (Homozygous four base pair deletion in exon 26 of the SPINK5 gene) presented with generalised erythema and scaling associated with marked pruritus. She had received treatment in the form of topical medications including emollients, corticosteroids, and a calcineurin inhibitor as well as systemic drugs including antihistamines, corticosteroids, acitretin (0.3 mg/kg/day for 2 months) and monthly doses of intravenous immunoglobulin (IVIG, 0.4 g/kg/day for 6 months) in the past (reported earlier) (1). The patient had minimal response to acitretin therapy, but showed a marked reduction in the erythema and scaling with IVIG. However, 6 months later, the child developed thrombosis of left sigmoid, and transverse sinus for which she was started on enoxaparin and IVIG was discontinued. Her past history was significant for growth hormone deficiency and exogenous Cushing's syndrome. On examination, the patient had central obesity with prominent striae. Cutaneous examination revealed generalised erythema and scaling in the form of ichthyosis linearis circumflexa (ILC) (Figure 1). Scalp hair was short and sparse and revealed trichorrhexis nodosa on light microscopy. The patient was planned for secukinumab therapy and relevant investigations were performed. To assess the efficacy of anti-IL17 therapy, mRNA expression of Th17 related pathway genes were checked at three different time points (Figure 3). Assessment of CD4^+^-T helper cell population in whole blood received from patient before and after anti-IL17A therapy (Figure 4).

**Figure 2:**
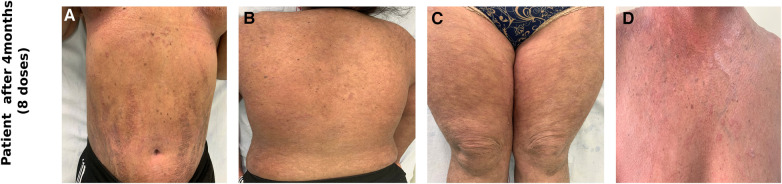
Clinical photographs of the patient after receiving 8 doses of subcutaneous secukinumab showing marked reduction in erythema and scaling over the abdomen (**A**), back (**B**), thighs (**C**), chest (**D**).

**Figure 3:**
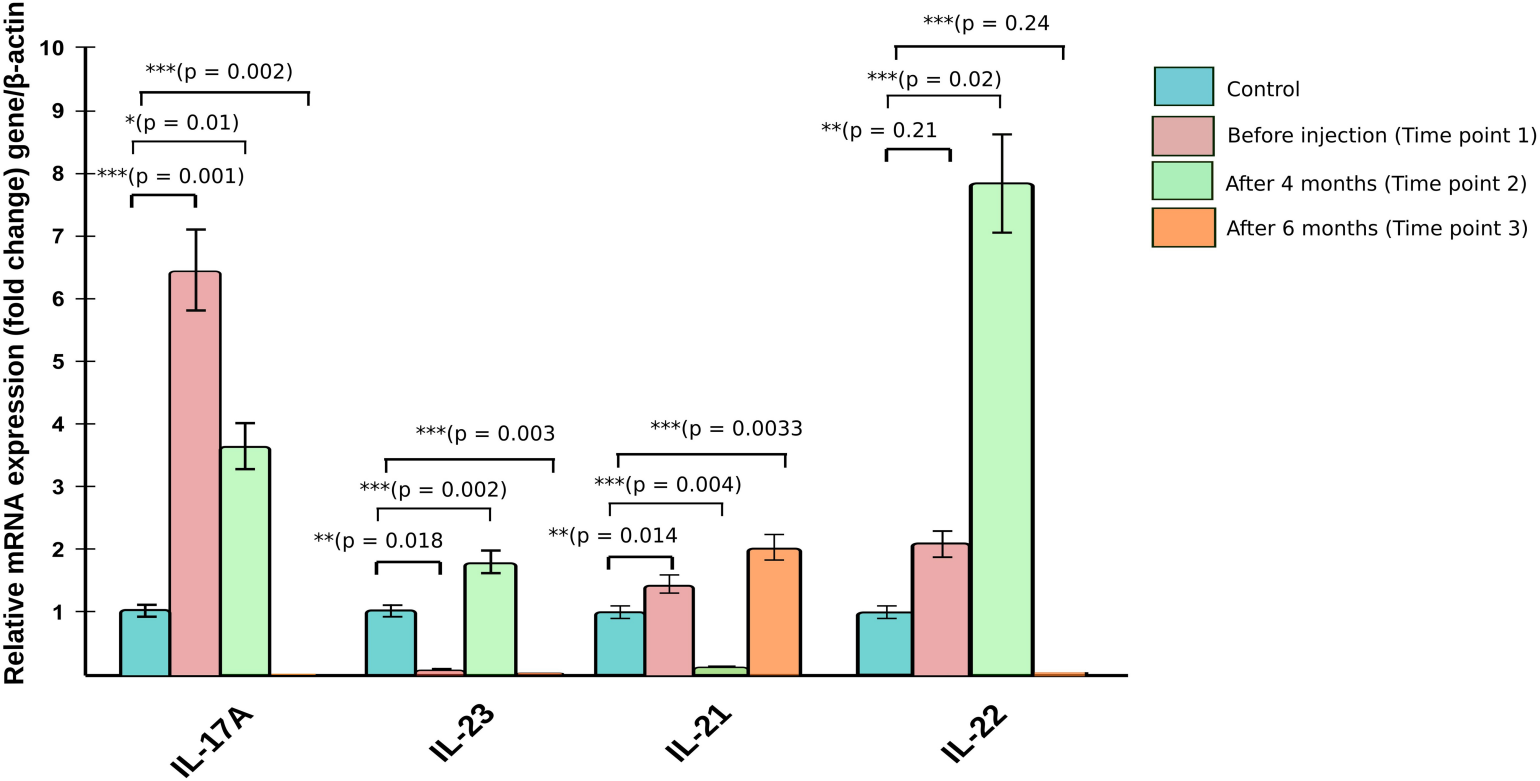
Relative mRNA expression of cytokine genes from patient before and after anti-IL17A therapy.

**Figure 4:**
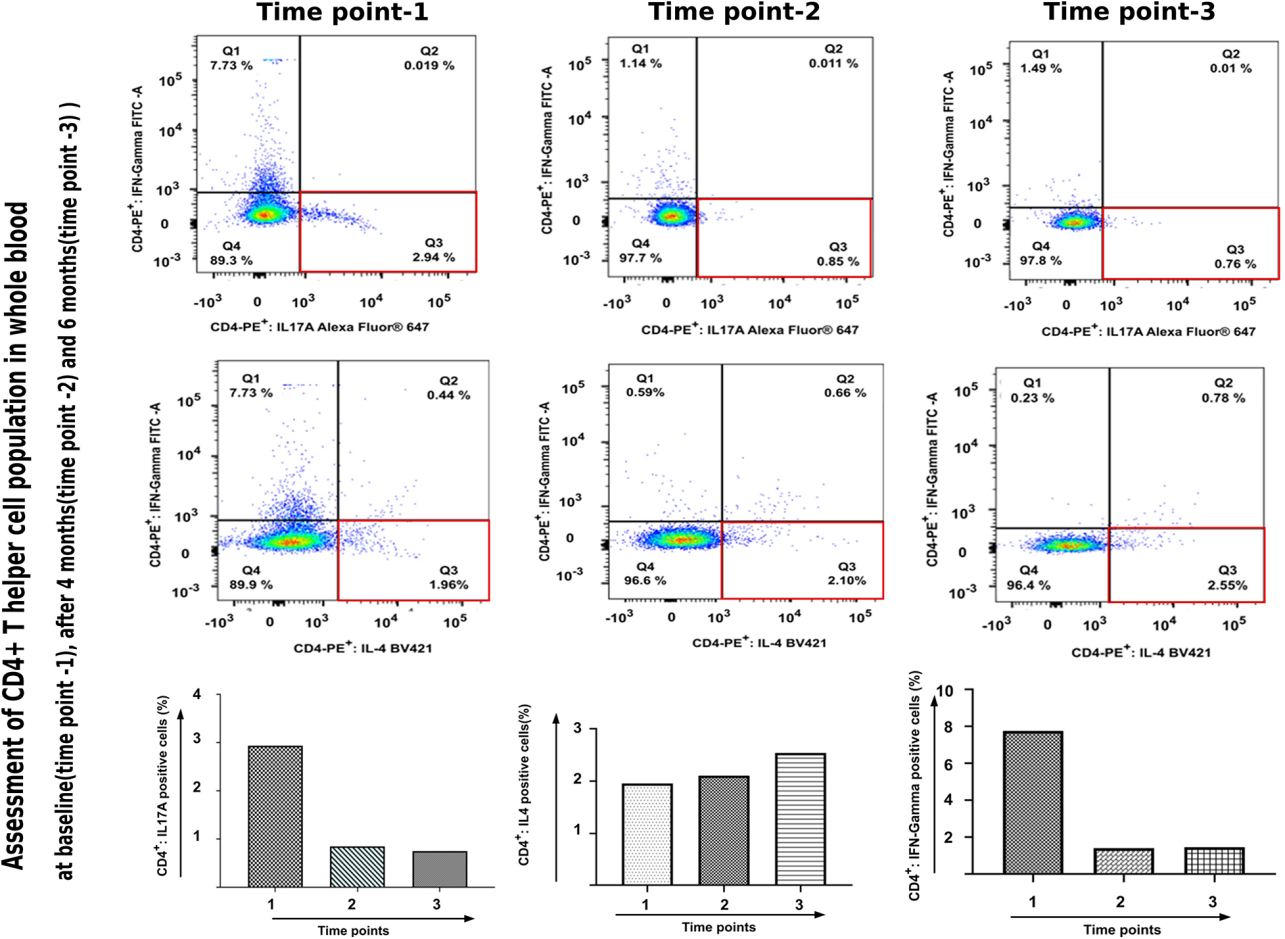
Assessment of CD4^+^-T helper cell population in whole blood received from patient before and after anti-IL17A therapy.

The blood counts and biochemistry were within normal limits; viral markers were non-reactive and chest x-ray did not show any abnormalities. Work-up for primary immunodeficiency disease (serum immunoglobulins, T-cell subtyping, nitroblue tetrazolium tests, complement levels) was also normal. Subsequently, the patient was administered subcutaneous 150 mg (weight of the patient being 53 kg)once a week for 5 doses followed by once a month. Significant improvement in disease severity in terms of pruritus as well as erythema and scaling could be observed after 8 doses (4 months) of secukinumab therapy (Figures 2A–2D). The ichthyosis severity score decreased considerably from 36 (pretreatment) to 18 after 4 months of treatment with a significant improvement in quality of life. Possible adverse event in the form of appearance of verruca vulgaris was observed after 4 doses of secukinumab therapy, however the same resolved without any active intervention within 2 months. No serious adverse events were reported. The relative mRNA levels of Th17 pathways were examined (Figure 3). The relative mRNA expression of the *IL17A* (6.45-fold; *p *=* *0.001)*, IL21* (1.45-fold; *p *=* *0.014)*,* and *IL22* (2.08-fold; *p *=* *0.21) cytokines were elevated at first time points (before first dose of injection). Significant decrease was observed in mRNA level of *IL17A* gene at second (3.66-fold; *p *=* *0.01) and third (0.0001-fold; *p *=* *0.002) time points. The mRNA level of *IL23* gene was low during first time point (0.11-fold, *p* = 0.018), unexpectedly increased during second time points (1.80-fold, *p *=* *0.002) and reduced after third time point (0.0010 fold, *p *=* *0.003). T-helper cell subtypes were assessed by flowcytometry techniques (Figure 4). There was gradual reduction in CD4^+^-IL17A positive cells (Th17 cells) from first time point to third time point (2.76%–0.76%). After second time point there was not much increase. CD4^+^-IL4 positive cell (Th2 cells) population was slightly increased in second (2.10%) and third time point (2.10%) as compared to first time point (1.96%). The reduction in CD4^+^-IFN-Gamma postive cells (Th1 cells) was sharp in second and third time point among all T-helper cell population. All together anti-IL17 therapy shows reduction in T-helper cells in the patients at three different time points.

## Discussion

NS is a rare autosomal recessive disorder characterized by a triad of congenital ichthyosiform erythroderma, hair shaft abnormalities, and atopic diathesis (elevated serum IgE) (2). The pathogenetic loss-of-function mutations in the serine protease inhibitor Kazal type 5 gene located on chromosome 5q31-32 encoding the lymphoepithelial Kazal-type related inhibitor leads to a reduction of serine protease inhibition in turn causing over desquamation of corneocytes.

According to the European Guidelines for the management of congenital ichthyosis, the treatment of NS involves use of topical emollients, keratolytics, glucocorticoids, calcineurin inhibitors, and calcipotriol. New therapeutic options involving narrowband ultraviolet B phototherapy, IVIG and anti TNF-α also appear to be effective as a treatment (3).

With the emerging evidence of 1l-17 driven inflammatory pathways playing a role in the pathogenesis of inherited ichthyosis and responsible for the cutaneous inflammation, interest has arisen in targeting this particular pathway for the treatment of ichthyosis (4, 5).

Secukinumab is a recombinant human monoclonal immunoglobulin G1 (IgG1)/*κ* antibody that selectively targets IL-17A and blocks its interaction with the IL-17 receptor. It has been used successfully in other proliferative skin disorders especially psoriasis where it effectively reduces the erythema and scaling by suppressing the IL-17 pathway. It is FDA approved for the treatment of moderate to severe plaque psoriasis, psoriatic arthritis and active ankylosing spondylitis. Recently, secukinumab has received FDA approval for the treatment of children (≥6 years) and adolescents with moderate to severe plaque psoriasis (6). There have been reports of marked cutaneous improvement in patients of NS with secukinumab therapy (7–10).

Our patient had already received multiple treatments in the past, however continued therapy with any of the drugs was not feasible either due to insufficient response or due to development of serious adverse events. Exhaustion of conventional therapies as well as promising results shown by secukinumab in patients with NS prompted us to initiate secukinumab therapy in this patient. The results were striking with marked cutaneous improvement of parameters including pruritus, erythema and ILC like scales. The patient continues to be maintained on monthly doses of secukinumab without any serious adverse events.

Our case further highlights the therapeutic potential of the interleukin-17 inhibitor drugs like secukinumab in patients with NS. However, long term studies on larger populations are needed to validate the findings of this study.

## Data Availability

The original contributions presented in the study are included in the article/Supplementary Material, further inquiries can be directed to the corresponding author.

## References

[1] Dabas G Mahajan R De D Handa S Kumar R Dayal D Managing syndromic congenital ichthyosis at a tertiary care institute-genotype-phenotype correlations, and novel treatments. Dermatol Ther. (2020) 33(6):e13816. 10.1111/dth.13816 32533806

[2] Wilkinson RD Curtis GH Hawk WA. Netherton’s disease; trichorrhexis invaginata (bamboo hair), congenital ichthyosiform erythroderma and the atopic diathesis. A histopathologic study. Arch Dermatol. (1964) 89:46–54. 10.1001/archderm.1964.01590250052010 14070837

[3] Mazereeuw-Hautier J Hernández-Martín A O’Toole EA Bygum A Amaro C Aldwin M Management of congenital ichthyoses: european guidelines of care, part two. Br J Dermatol. (2019) 180(3):484–95. 10.1111/bjd.16882 29897631

[4] Paller AS Renert-Yuval Y Suprun M Esaki H Oliva M Huynh TN An IL-17-dominant immune profile is shared across the major orphan forms of ichthyosis. J Allergy Clin Immunol. (2017) 139(1):152–65. 10.1016/j.jaci.2016.07.019 27554821 PMC8033419

[5] Barbieux C Bonnet des Claustres M Fahrner M Petrova E Tsoi LC Gouin O Netherton syndrome subtypes share IL-17/IL-36 signature with distinct IFN-α and allergic responses. J Allergy Clin Immunol. (2022) 149(4):1358–72. PMID: . 10.1016/j.jaci.2021.08.024 34543653

[6] Novartis Cosentyx receives FDA approval for treatment of children and adolescents with moderate to severe plaque psoriasis. Novartis. Available at: https://www.novartis.com/news/media-releases/novartis-cosentyx-receives-fda-approval-treatment-children-and-adolescents-moderate-severe-plaque-psoriasis (Cited January 13, 2022).

[7] Blanchard SK Prose NS. Successful use of secukinumab in netherton syndrome. JAAD Case Rep. (2020) 6(6):577–8. 10.1016/j.jdcr.2020.04.025 32518812 PMC7270530

[8] Luchsinger I Knöpfel N Theiler M Bonnet des Claustres B Barbieux C Schwieger-Briel A Secukinumab therapy for netherton syndrome. JAMA Dermatol. (2020) 156(8):907–11. 10.1001/jamadermatol.2020.1019 32459284 PMC7254452

[9] Gan C King E Orchard D. Secukinumab use in the treatment of netherton’s syndrome. Australas J Dermatol. (2022) 63(3):365–7. 10.1111/ajd.13880 35622930

[10] Pontone M Giovannini M Filippeschi C Oranges T Pedaci FA Mori F Biological treatments for pediatric netherton syndrome. Front Pediatr. (2022) 10:1074243. 10.3389/fped.2022.1074243 36619513 PMC9822572

